# Advances in radiotherapy and immunity in hepatocellular carcinoma

**DOI:** 10.1186/s12967-023-04386-y

**Published:** 2023-08-04

**Authors:** Yuhan Yang, Liting Xiong, Mengyuan Li, Ping Jiang, Junjie Wang, Chunxiao Li

**Affiliations:** 1 Department of Radiation Oncology, Peking University Third Hospital, Beijing, 100191 China; 2 Institute of Medical Technology, Peking University Health Science Center, Beijing, 100191 China

**Keywords:** Hepatocellular carcinoma, Radiotherapy, Immunotherapy, Radioimmunotherapy

## Abstract

Primary liver cancer is one of the most common malignant tumours worldwide; it caused approximately 830,000 deaths in 2020. Hepatocellular carcinoma (HCC) is the most common type of primary liver cancer, accounting for over 80% of all cases. Various methods, including surgery, chemotherapy, radiotherapy, and radiofrequency ablation, have been widely used in the treatment of HCC. With the advancement of technology, radiotherapy has become increasingly important in the comprehensive treatment of HCC. However, due to the insufficient sensitivity of tumour cells to radiation, there are still multiple limitation in clinical application of radiotherapy. In recent years, the role of immunotherapy in cancer has been increasingly revealed, and more researchers have turned their attention to the combined application of immunotherapy and radiotherapy in the hope of achieving better treatment outcomes. This article reviews the progress on radiation therapy in HCC and the current status of its combined application with immunotherapy, and discusses the prospects and value of radioimmunotherapy in HCC.

## Introduction

Primary liver cancer is the third leading cause of cancer-related mortality worldwide, accounting for 8.3% of total cancer deaths, following lung cancer (18.0%) and colorectal cancer (9.4%). In 2020, it accounted for a total of 830,000 deaths and 906,000 new cancer cases globally, and it has significantly higher incidence and mortality rates in males than in females [1]. Hepatocellular carcinoma (HCC) is the main type of primary liver cancer, and China is a high-risk region for HCC [2]. Chronic viral infection is the major factor leading to HCC, and other risk factors include exposure to aflatoxin, excessive alcohol consumption, obesity, etc. [3]. The treatment options for HCC vary depending on factors such as tumour stage, and location [4]. For early-stage HCC patients, local resection and liver transplantation are the preferred treatment options, with 5-year survival rates ranging from 70 to 80% [5]. Radiofrequency ablation is the preferred alternative method for unresectable early-stage HCC patients, and transarterial chemoembolization is the standard choice for intermediate-stage HCC patients [6]. However, most HCC patients are diagnosed at an advanced stage, at which point they have lost the opportunity for local curative treatment [7]. Although the combination of the anti-PD-L1 antibody atezolizumab and the anti-VEGF antibody bevacizumab has significantly improved the survival of advanced-stage patients, drug resistance remains a pressing issue that needs to be addressed [8].

With the continuous advancement of technology, the role of radiotherapy has been increasingly recognized, and it has been widely used as an integral part of comprehensive treatment for HCC [9]. Precision radiotherapy techniques, such as stereotactic body radiotherapy (SBRT), accurately deliver high doses to the target area while reducing radiation damage to surrounding normal organs and tissues; these approached, show excellent local control in early-stage HCC patients who are not eligible for surgery or ablation treatment and play an important role as bridging therapy options for liver transplantation [10–12]. Radiation sensitivity is a primary barrier that limits the efficacy of tumour treatment, and the mechanisms affecting radiation sensitivity in HCC are complex and diverse. Combination with other treatment modalities may offer more clinical benefits for patients [13]. The liver harbours the largest population of tissue-resident macrophages, known as Kupffer cells, as well as various other types of infiltrating lymphocytes, creating a unique tumour microenvironment in HCC [14]. Previous studies have also shown that radiotherapy can enhance the immunogenicity of tumours, indicating the great potential of combining radiotherapy with immunotherapy in the comprehensive treatment of HCC [15]. This review summarizes the progress on radiotherapy in HCC and the current status of radiotherapy combined with immunotherapy, and discusses the future prospects and research value of this combination treatment approach in HCC.

## Current status of radiotherapy for HCC

As a common malignant tumour, primary liver cancer ranks third in global cancer mortality, following lung cancer and colorectal cancer. According to statistics, there were approximately 906,000 new cases and 830,000 deaths from primary liver cancer worldwide in 2020, and the incidence and mortality rates in males were 2–3 times higher than those in females in most regions [1]. Data from the National Cancer Center (NCC) of China show that primary liver cancer ranks fourth among malignant tumours in China in terms of incidence and second in terms of mortality rate, imposing a heavy disease burden on the country [16]. HCC accounts for approximately 80–90% of primary liver cancer cases, while intrahepatic cholangiocarcinoma (CCA) and other rare types account for 10–15%. The main risk factors for the development of HCC include infection with hepatitis B virus (HBV) or hepatitis C virus (HCV), chronic toxin exposure (such as aflatoxin), alcohol abuse, obesity, etc., with the first two being the predominant factors in China [17]. For early-stage HCC patients, surgical resection is the preferred curative treatment, but most patients are diagnosed at advanced stages, and only a few patients have the opportunity to undergo radical resection [18]. Local treatment strategies, including radiofrequency ablation or microwave ablation, are mostly used to control tumours with a diameter smaller than 4 cm, and their efficacy is limited in large tumours or tumours near major blood vessels or with vascular involvement [19]. Systemic treatments such as sorafenib, lenvatinib, and other targeted therapies have improved the prognosis of advanced HCC, but primary or acquired drug resistance still occurs in a significant proportion of patients [20].

In recent years, radiotherapy has gradually become widely used as an important local therapy for HCC and has become an essential component of its comprehensive treatment. Early limitations in technology, such as limited accuracy and wide irradiation range of radiotherapy, resulted in suboptimal treatment outcomes, high toxicity, and inadequate dosages. With the emergence of precision radiotherapy techniques, these issues have gradually been overcome [21]. With steep dose gradients and precise target coverage, SBRT is an important local treatment method for HCC, not only demonstrating excellent local control in the primary tumour, but also serving as a safe and effective therapy for bridging to liver transplantation and reducing tumour volume [22]. A study of 297 HCC patients without vascular invasion included in the research tracked overall survival (OS), liver function, alpha-fetoprotein, and other indicators within five years after SBRT. The results showed that SBRT provided high local control and long-term survival for a significant proportion of HCC patients who were not eligible for or had adverse effects with standard local regional treatment [23]. For HCC patients with microvascular invasion at the surgical margin, SBRT can also provide a safe adjuvant treatment option to prevent local recurrence and improve disease-free survival [24]. In a study comparing the local control rates and OS of SBRT and transarterial chemoembolization (TACE) in patients with medium-sized HCC tumours (diameter 3–8 cm), SBRT showed better local control rates and OS than TACE, particularly for recurrent HCC patients [25]. Another study showed that for 1–2 tumours, SBRT can be a safe alternative to TACE, but no significant difference in OS was observed between the two [26]. A retrospective cohort study found that SBRT had a lower risk of local recurrence than radiofrequency ablation (RFA); subgroup analysis also showed that SBRT was associated with better local control rates for small tumours (diameter ≤ 3 cm) irrespective of location; for tumours located below the diaphragm and tumours progressing after TACE, SBRT showed lower toxicity than RFA. Therefore, SBRT may be an effective alternative to RFA for unresectable HCC [27]. Liver transplantation is an important life-saving option for some HCC patients, but there is a risk of tumour progression or recurrence during the waiting process for a liver transplant [4]. Researchers have compared the safety and effectiveness of SBRT, TACE, and RFA as bridging therapies for liver transplantation, and the results showed that SBRT can be a good alternative to conventional bridging therapies [28]. Patients with advanced HCC with macrovascular invasion usually have a poor prognosis, and the response to sorafenib and the survival benefit are still unsatisfactory [29]. In a prospective randomized controlled trial, researchers compared the treatment outcomes of patients receiving sorafenib alone and patients receiving TACE plus SBRT. The results showed that the progression-free survival rate at week 12 was significantly higher in the TACE-SBRT group than in the sorafenib group, and the median progression-free survival and OS rates at week 24 were notably higher in the TACE-SBRT group than in the sorafenib group. Therefore, TACE plus SBRT may be a better treatment option than sorafenib alone for advanced HCC patients with macrovascular invasion [30]. In addition to SBRT, high-dose fractionated proton beam therapy (HPT) is also a treatment option for HCC. A phase II study evaluated the efficacy and safety of HPT in HCC. The results showed a 2-year local control rate of 94.8% and a 2-year overall survival rate of 63.2%, indicating that the application of HPT in HCC is safe and effective [31].

However, limited sensitivity of tumour to radiotherapy is one of the important factors that results in lower-than-expected treatment outcomes [32–34]. A study observed the survival fraction of HCC cell lines in 16 patients and classified them into sensitive, moderately sensitive, and radioresistant groups. The results showed that the radiosensitivity of the cell lines was mainly distributed in the moderately sensitive group (43.75%) and the radioresistant group (37.5%), with only approximately one-fifth of the cell lines (18.75%) classified as sensitive [35]. Although various solid tumours, including cervical cancer, have been treated with concurrent chemotherapy to enhance sensitivity to radiotherapy, the effects of traditional radiosensitizers such as mitomycin and 5-FU in HCC seem to be less satisfactory. Therefore, improving the radiosensitivity of HCC to enhance treatment outcomes has become a current research hotspot [36].

**Fig. 1:**
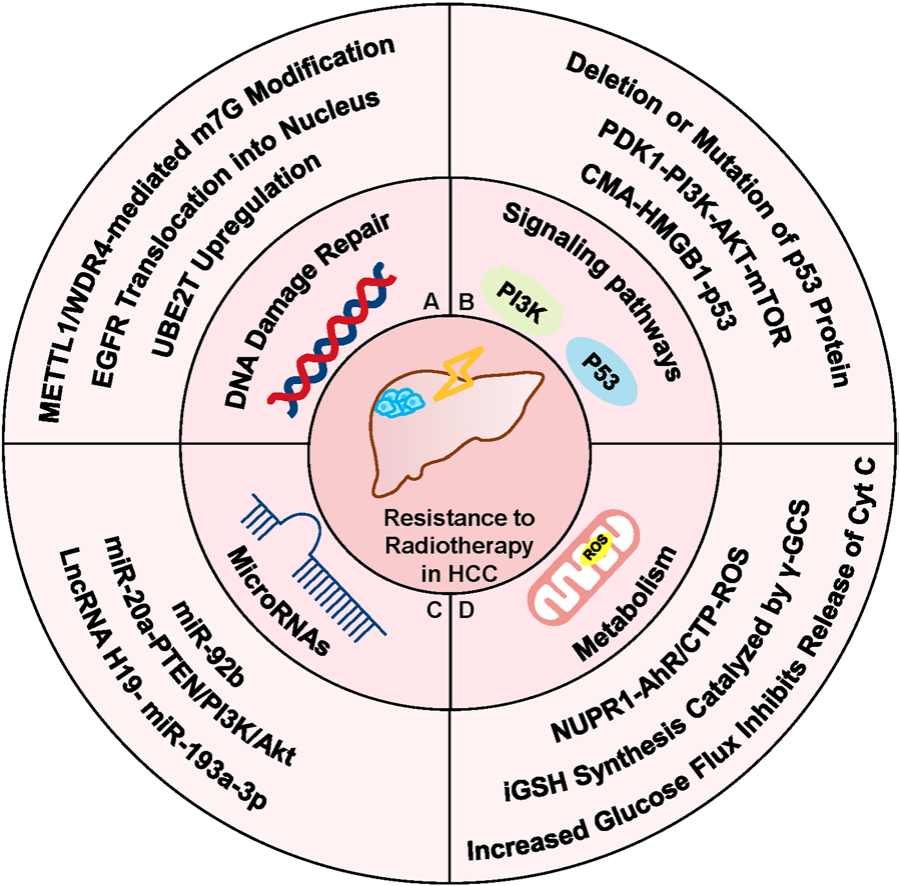
Molecular mechanisms of resistance to radiotherapy in HCC. **A** DNA damage repair is an important contributor to radiation resistance. Both METTL1/WDR4-mediated modification of m7G tRNA and EGFR mutations enhance the efficiency of DNA double-strand break (DSB) repair in the nonhomologous end-joining (NHEJ) pathway by augmenting the activity of the catalytic subunits of DNA-dependent protein kinases (DNA-PKcs). Upregulation of the ubiquitin-conjugating enzyme E2T (UBE2T) in HCC cells plays a similar role. **B** Changes in multiple signalling pathways in HCC cells affect their sensitivity to radiation. Deletion and mutation of p53 both decrease the radiosensitivity of HCC, and chaperone-mediated autophagy (CMA) impairs the efficacy of radiotherapy by downregulating p53. The effect of phosphoinositide-dependent protein kinase-1 (PDK1) on HCC radiosensitivity is mediated by activation of the PI3K/AKT/mTOR signalling pathway, which inhibits DNA damage repair. **C** MicroRNAs (miRNAs) are involved in the regulation of radiosensitivity in HCC by affecting cell proliferation and cell cycle. MiR-92b, mir-20a and miR-193a-3p confer radiation resistance to HCC cells in different ways. **D** The role of metabolism in radiosensitivity cannot be ignored. Glucose addiction in HCC cells promotes phospholipid synthesis, which inhibits cytochrome c release and reduces radiation-induced apoptosis. γ-Glutamylcysteine synthetase (γ-GCS) and nuclear protein 1 (NUPR1) inhibit cellular oxidative stress by producing GSH and modulating the AhR/CTP signalling axis, respectively, thereby enhancing cell viability after radiotherapy

The DNA damage response (DDR) induced by radiation is key to the tumour-killing effect of radiotherapy, with DNA double-strand breaks (DSBs) being the most lethal form of damage, which is repaired mainly through homologous recombination (HR) or nonhomologous end joining (NHEJ) pathways [37]. These DNA damage repair responses confer protection to normal tissues, while providing resistance to radiation in tumour cells [38]. As one of the most prevalent RNA modifications, N^7^-methylguanosine (m^7^G) plays an important role in regulating RNA processing, function, and gene expression and is catalysed mainly by methyltransferase-like 1 (METTL1) and WD repeat domain 4 (WDR4) proteins [39]. Studies have revealed that METTL1/WDR4-mediated modification of m^7^G tRNA can significantly promote HCC progression, and this promotion effect is associated with cell cycle regulation and EGFR signalling pathways. Knockdown of METTL1 inhibits the translation of cell cycle protein A2, which subsequently leads to G2/M cell cycle arrest. It also reduces the mRNA translation of EGFR and VEGFA signalling pathway components, thereby inhibiting HCC proliferation and metastasis [40]. Studies have explored the interaction between tRNA modification and radiation resistance and found that the RNA methyltransferase METTL1 is closely related to the radiation resistance in HCC. The results showed that tRNA modification catalysed by METTL1 can promote DNA double-strand break repair, leading to insensitivity of HCC to radiotherapy. The molecular mechanism is that the m7G tRNA modification mediated by METTL1 is able to upregulate the translation of DNA-dependent protein kinase catalytic subunits (DNA-PKcs) or DNA ligase IV required for DNA damage repair, which improves the efficiency of DSB repair in the NHEJ pathway. In addition, clinical data have also shown that high expression of METTL1 is significantly correlated with a poor prognosis in HCC patients after radiotherapy [41]. Epidermal growth factor receptor (EGFR) is a member of the subfamily of membrane-bound receptor tyrosine kinases (RTKs), which can phosphorylate intracellular tyrosine residues, activate downstream signalling pathways (e.g., the RAS-RAF-MEK-ERK, JAK-STAT, and PI3K-AKT pathways) and regulate a wide range of biological processes [42]. EGFR mutations have been shown to promote the development and progression of various tumours [43–45]. Despite the promising results of drugs targeting EGFR in tumours, their complex drug resistance cannot be ignored [46]. Studies have shown that the activation of EGFR significantly inhibits the response of HCC to the tyrosine kinase inhibitor lenvatinib, and this resistance is overcome after inhibition of the EGFR -STAT3-ABCB1 pathway [47, 48]. In addition, mutations in EGFR are also closely associated with radiotherapy resistance, with proliferation of tumour cells, DNA damage repair, hypoxia, and tumour metastasis formation being the four mechanisms, and DNA damage repair occupying an important position [49]. DNA-PKcs is a key enzyme in the NHEJ pathway that can be used to facilitate DNA damage repair after exposure to radiation [50]. However, radiation can promote the translocation of EGFR to the nucleus or promote the activation of EGFR directly, which could increase the activity of DNA-PKcs and promote DSB repair in the NHEJ pathway, making radiation ineffective [38]. Another study found that ubiquitin-conjugating enzyme E2T (UBE2T) is upregulated in HCC, and HCC patients with higher levels of UBE2T are less sensitive to radiotherapy. This is because the presence of radiation resistance is due to monoubiquitination of H2AX/γH2AX mediated by UBE2T, which promotes activation of checkpoint kinase 1 (CHK1) and provides sufficient time for radiation-induced DNA repair [51] (Fig. 1A). As p53 is a key tumour suppressor molecule, p53 mutation occurs in various types of malignancies [52]. Mutant(mut) p53 loses the inhibitory function of wild-type(wt) p53 and promotes the proliferation, invasion, metastasis, and metabolic reprogramming of tumour cells, which are closely associated with the development of most malignancies [53]. Radiotherapy-induced DNA damage activates the p53 signalling pathway, and p53 mutations also have an impact on the biological effects of ionizing radiation, which are often associated with radiation resistance [54–56]. This mechanism was first identified in head and neck squamous cell carcinoma cell lines and subsequently confirmed in bladder tumours [57, 58]. One study compared the radiosensitivity of three HCC cell lines: MHCC97L mutp53 cells, Hep3B p53 null cells, and HepG2 wtp53 cells. The results showed that the radiosensitivity of MHCC97L cells was much lower than that of the other two cell lines, indicating that deletion or mutation of the p53 protein is closely related to the radioresistance of HCC cells [59]. Chaperone-mediated autophagy (CMA) is a type of autophagy, and previous studies have shown that CMA can reduce the level of accumulated mutant p53 protein [60]. Researchers have found that radiation-induced activation of CMA can degrade the high-mobility group box 1 (HMGB1) protein and downregulate p53, thereby conferring radiation resistance to HCC cells [61]. The phosphatidylinositol-3 kinase (PI3K)/protein kinase B (PKB, AKT)/mammalian target of rapamycin (mTOR) signalling pathway is involved in the regulation of a variety of cellular activities, such as proliferation, metabolism, apoptosis, and autophagy, and aberrant activation of this pathway occurs in approximately 50% of HCC patients [62]. One study explored the effect of phosphoinositide-dependent protein kinase-1 (PDK1) on radiosensitisation in HCC. The results showed that PDK1 could activate the PI3K/AKT/mTOR signalling pathway, thereby inhibiting DNA damage repair and making HCC radiation therapy ineffective [62]. In contrast, PKI-587, a dual PI3K/mTOR inhibitor, was able to increase the sensitivity to radiotherapy [63] (Fig. 1B).

MicroRNAs (miRNAs) are endogenous noncoding RNAs that play a regulatory role by inhibiting the translation of target messenger RNAs (mRNAs), and their mutation or misexpression is closely associated with a wide range of malignancies [64]. miRNA dysregulation plays an important role in the development of HCC and is involved in the regulation of radiosensitivity in HCC [65]. This regulatory role involves various mechanisms such as DNA damage repair, cell cycle regulation, and apoptosis [66]. Some miRNAs positively regulate the radiosensitivity of HCC cells. MiR-146a-5p could restrict proliferation and promote apoptosis to improve radiosensitivity by activating DNA damage repair pathways and repressing replication protein A3 (RPA3) expression [67]. The formation of γ-H2AX upon phosphorylation of histone 2AX (H2AX) is an important indicator of DSBs [68]. Overexpression of RAD21, an important protein for DNA damage repair and homologous recombination, reduces γ-H2AX levels and decreases the efficiency of radiotherapy. MiR-320b was able to inhibit RAD21 expression by targeting the RAD21 3'-UTR, thereby increasing the radiosensitivity of HCC [69]. The expression of miR-621 is significantly reduced in HCC tissues compared to normal tissues, and the SET domain bifurcated 1 (SETDB1) gene is a direct target gene of miR-621. It was found that miR-621 could inhibit the expression of SETDB1 by targeting its 3' UTR, and the miR-621/SETDB1 signalling axis further activated the p53 signaling pathway to enhance the radiosensitivity of HCC [70]. However, many miRNAs inhibit the efficacy of radiotherapy for HCC. A study exploring the effects of miR-193a-3p on HCC cell lines found that miR-193a-3p could enhance cellular resistance to radiation by promoting DNA double-strand break repair, and the long-stranded noncoding RNA (lncRNA) H19/miR-193a-3p signaling pathway is a promising therapeutic target in HCC radiotherapy [71]. MiR-92b has also been found to affect the response of HCC to radiotherapy. MiR-92b is overexpressed in both HCC tissues and cell lines, and is associated with poor patient prognosis. Overexpression of miR-92b promotes tumour cell proliferation, inhibits apoptosis, and ameliorates radiation-induced cell cycle arrest, thereby reducing the sensitivity of HCC cells to radiation [72]. Additionally, both in vivo and in vitro experiments also demonstrated that mir-20a overexpression in HCC could activate the PTEN/PI3K/Akt signalling pathway, which conferred greater radiation resistance to tumour cells [73] (Fig. 1C). Metabolism is closely associated with the sensitivity of HCC to radiation. Researchers screened these cell lines with radiation resistance and found that HCC cells with radiation resistance showed increased dependence on glucose after studying proteomics, metabolomics, and metabolic flux. Increased glucose flux promotes the synthesis of glucose into phospholipids, the accumulation of which inhibits the release of cytochrome c, reducing radiation-induced cell apoptosis. Glucose addiction in HCC cells is dependent on HIF-1α, and mTORC1 mediates the radiation resistance of HCC by enhancing the translation of HIF-1α and SREBP1 [74]. Moreover, γ-glutamylcysteine synthetase (γ-GCS) has been identified as a possible target to overcome radioresistance in HCC. Researchers have found that γ-GCS is the rate-limiting enzyme that regulates GSH biosynthesis and that γ-GCS can enhance HCC radiosensitivity by catalysing endogenous GSH synthesis [75]. Nuclear protein 1 (NUPR1) also plays a key role in regulating oxidation‒reduction reactions in vivo and is able to inhibit reactive oxygen species (ROS) production and oxidative stress through the AhR/CTP signalling axis, thereby increasing cell viability during radiotherapy [76] (Fig. 1D).

Therefore, considering the radioresistant status of HCC with multiple complex mechanisms, combining radiotherapy with other treatment modalities to enhance the body's antitumour immune response seems to be a more effective approach.

## Effects of radiotherapy on immunity in HCC

The tumour microenvironment (TME) consists of noncancerous host cells and other noncellular components present in the tumour, and the continuous interaction between tumour cells and the TME plays a critical role in tumour initiation, progression, metastasis, and response to treatment [77]. In 2006, Dr. Robert Schreiber proposed the concept of "immune editing," which describes how tumour cells evade immune responses and even "reprogram" certain immune cells to promote tumour growth [78]. This process of immune editing occurs almost every time a tumour is present, resulting in a highly suppressed TME [79]. One mechanism of immune escape is achieved by tumour cells inducing and recruiting immune suppressive cells and increasing the expression of various immune inhibitory molecules [80]. The liver is an important immune organ enriched with a large number of innate immune cells, including natural killer cells (NK cells), natural killer T cells (NKT cells), and T cell receptor γδ (TCR γδ) T cells, of which NK cells in the liver can also be referred to as pit cells [81]. Studies have shown that pit cells in the liver have higher cytotoxicity against tumour cells than NK cells in the spleen and peripheral blood. This may be due to the upregulation of tumour necrosis factor-related apoptosis-inducing ligand (TRAIL) expression in pit cells [82]. In addition to an abundance of lymphocytes, the liver contains a large number of liver-specific immunoreactive cells, such as Kupffer cells, liver sinusoidal endothelial cells (LSECs), and hepatic stellate cells (HSCs) [83]. Both LSECs and HSCs can perform antigen-presenting functions in the liver. In contrast, LSECs can promote immune tolerance by secreting IL-10 and a variety of adhesion molecules to retain activated T cells in the hepatic sinusoids, including intercellular adhesion molecule-1 (ICAM-1), vascular cell adhesion molecule-1 (VCAM-1), and vascular adhesion protein-1 (VAP-1) [84]. While presenting antigens, HSCs can also inhibit T-cell activation by expressing PD-L1 [85]. Thus, the unique immune microenvironmental characteristics of the liver promote the occurrence of HCC and immune tolerance.

Research has revealed that major immunosuppressive cells associated with HCC immune escape include tissue-resident macrophages (mainly Kupffer cells), monocyte-derived macrophages, regulatory T cells (Tregs), and myeloid-derived suppressor cells (MDSCs) [86]. Kupffer cells are the most abundant population of tissue-resident macrophages in the liver and play a key role in immune suppression in the liver. Previous in vivo studies have shown that sustained and dysregulated chronic inflammation in the liver is carcinogenic, and Kupffer cells can drive tumour progression and metastasis in HCC, a process that is associated with their response to and activation of inflammatory signalling pathways. In the context of persistent chronic inflammation, activated Kupffer cells release C–C motif chemokine 2(CCL2) while recruiting monocytes and MDSCs from blood vessels and promoting HCC progression [87, 88]. The presence of tumour-associated macrophages (TAMs) is associated with a poor prognosis in HCC, particularly when TAMs are skewed towards the M2 polarization phenotype [89]. TAMs in HCC can secrete various cytokines and chemokines, such as IL-1β, IL-6, TNF, CCL2, and CXCL10, promoting tumour cell proliferation and NF-κB-mediated protection against cancer cell apoptosis, which is associated with a poor prognosis in HCC patients. TAMs can also produce vascular endothelial growth factor (VEGF), platelet-derived growth factor (PDGF), fibroblast growth factor (FGF), and other factors to support tumour tissue proliferation and growth [90, 91]. Treg cells, are an important subset of immunosuppressive cells, and their ratio to CD8 + T cells is an important prognostic indicator for cancer patients [92]. Compared with nontumour areas, the TME of HCC has significantly more CD4 + CD25 + FoxP3 + Treg cells and significantly fewer CD8 + T cells; and CCL2 and CCL5 produced by tumour cells play an important role in regulating this phenotype At this high level, Treg cells can impair the effector function of CD8 + T cells, significantly reducing the expression of granzyme A, granzyme B, and perforin in infiltrating CD8 + T cells, and promoting disease progression in HCC patients [93, 94]. Increasing evidence suggests that MDSCs also play an important role in the development of HCC, CCL2 and CCL5 from tumour cells and facilitate infiltration of MDSCs into the TME [95]. The level of MDSCs in the circulation of HCC patients is increased, and this high level is also associated with tumour progression and a poor prognosis in patients [96].

**Fig. 2:**
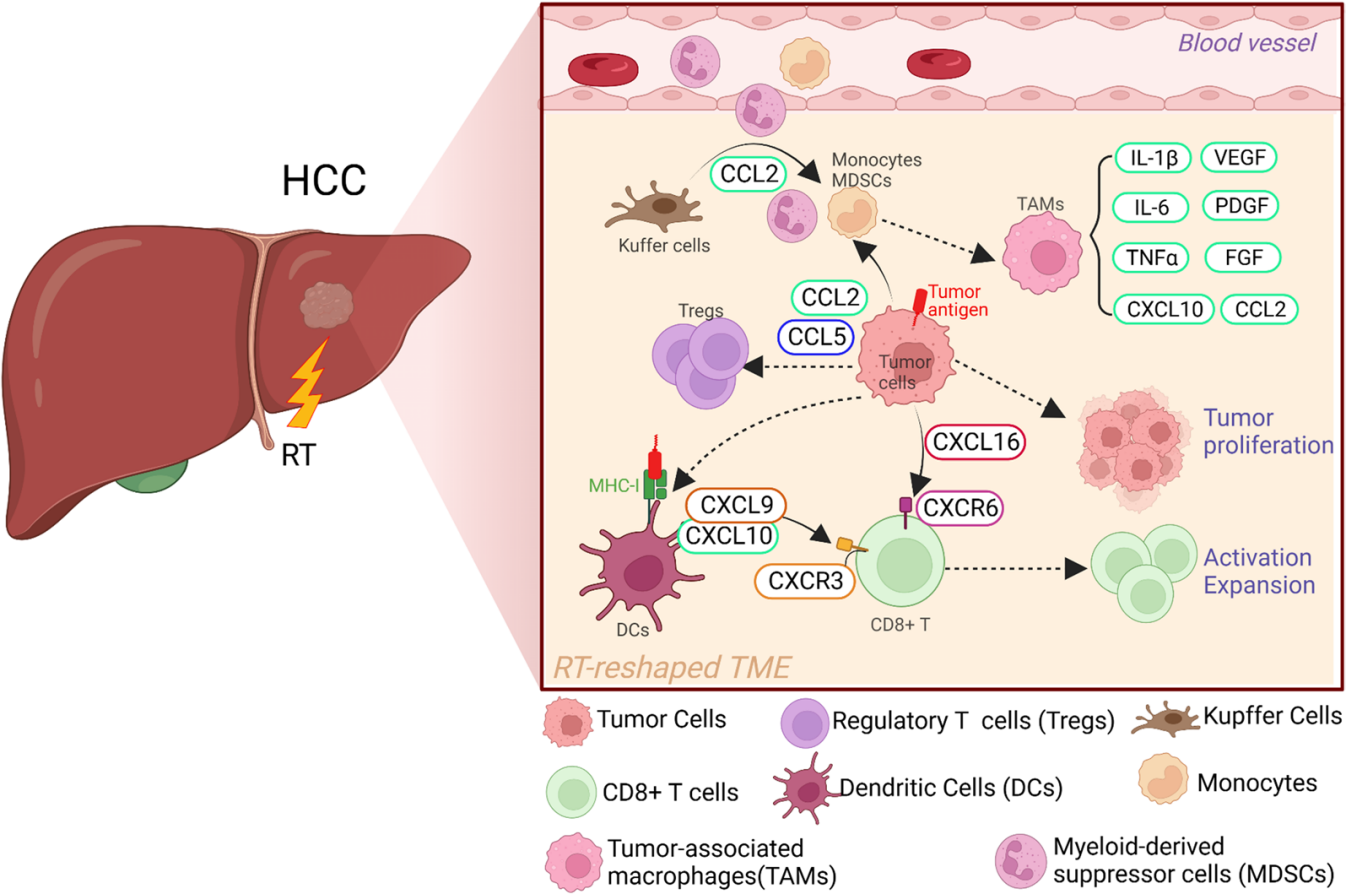
Changes in the TME of HCC before and after radiotherapy. TME of HCC before radiotherapy. (1) In the context of persistent chronic inflammation, activated Kupffer cells (KCs) release CCL2 while recruiting inflammatory monocytes and MDSCs and promoting HCC progression. (2) Tumour cell production of CCL5 and CCL2 promotes the infiltration of MDSCs and Tregs, which in turn inhibits the production of CD8+ T cells and immunosuppressive transforming growth factor β (TGF-β). (3) M2-like TAMs secrete interleukin 1β(IL-1β), IL-6, TNF, CCL2, and CXCL10, which promote the proliferation of tumour cells and are associated with poor patient prognosis. Radiotherapy reshapes the TME of HCC. (1) Radiotherapy induces the upregulated expression of MHC-I on the surface of tumour cells, which promotes the maturation of DCs and the presentation of tumour-associated antigens. Mature DCs release CXCL9 and CXCL10 to promote the infiltration of CD8+ T-cells in the TME. (2) Radiotherapy induces tumour cells to secrete CXCL16, which further promotes the activation and expansion of CD8+ T-cells

Radiotherapy, as one of the main modalities for the first-line treatment of multiple solid tumours, has a direct and significant impact on the tumour stroma, blood vessels, and immune cells [97]. Radiotherapy can upregulate major histocompatibility complex I (MHC-I) molecules on the surface of tumour cells, promote the maturation of dendritic cells (DCs) and presentation of tumour-associated antigens, and enhance the secretion of cytokines required for T-cell infiltration, such as chemokine (C–X–C motif) ligand 9 (CXCL9), CXCL10, and CXCL16, from DCs and tumour cells, which are beneficial for the cytotoxic activity and expansion of CD8 + T cells [98] (Fig. 2). Grassberger et al. conducted a prospective study on the levels of circulating lymphocytes in HCC patients receiving HPT. The results showed that the increase in CD8+ CD25+ T-cell levels during radiotherapy was associated with prolonged OS in HCC patients [99]. Studies have revealed that the levels of MDSCs in the body of HCC patients significantly decreased after radiotherapy, and this decrease was negatively correlated with overall survival time. In multivariate analysis, only posttreatment MDSC levels and Child‒Pugh classification were correlated with the prognosis of HCC patients, indicating that MDSC suppression achieved after radiotherapy may improve the prognosis of HCC patients [100]. Another study showed that high-dose fractionated radiation therapy of the primary site of transplanted tumours in a mouse HCC model could exert a potent abscopal effect on other sites of the same tumour. This abscopal effect may be achieved by reducing the levels of MDSCs in the body after radiotherapy [101]. Therefore, radiotherapy can have many positive effects on the immune microenvironment, reshaping the tumour microenvironment in multiple ways while killing tumour cells, providing strong support for the development of studies on radiation-immunotherapy strategies.

## The current status of combined application of radiotherapy and immunotherapy in HCC

**Fig. 3:**
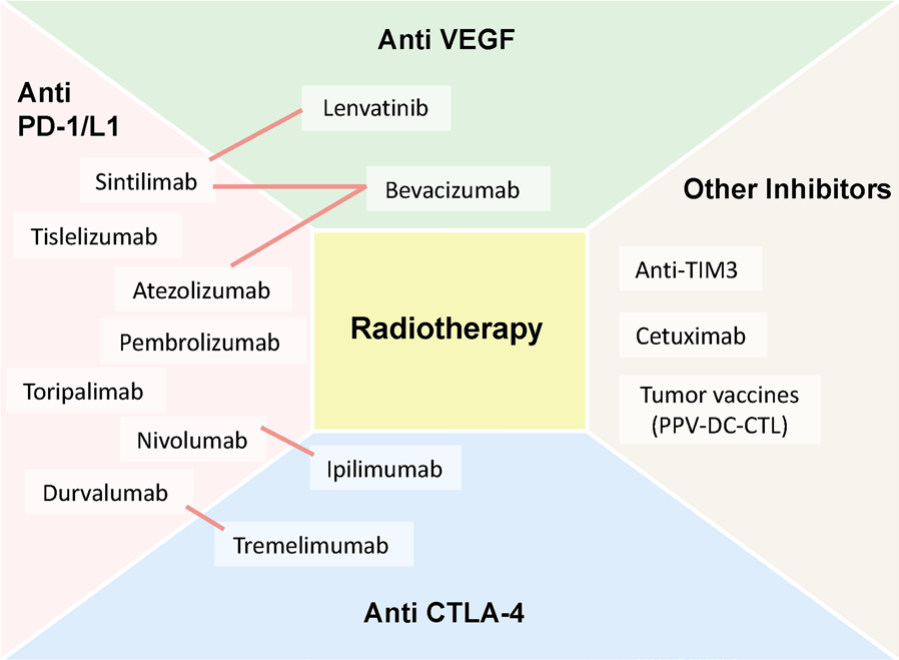
Combination treatments of radiotherapy and immunotherapy for HCC under investigation. Combination strategies reported or in ongoing investigations are presented, and lines demonstrate combinations of multiple immunotherapies. *PD-1/L1* programmed cell death 1/ ligand 1; *CTLA-4* cytotoxic T lymphocyte-associated antigen 4; *TIM3* T-cell immunoglobulin and mucin-domain containing molecule 3; *VEGF* vascular endothelial growth factor

However, radiotherapy still has certain limitations in the management of advanced HCC. Local tumour control achieved by radiotherapy does not necessarily translate into long-term survival for patients, as it cannot address the risk of liver dysfunction and metastasis or progression in other organs. Therefore, the combination of local treatment and systemic treatment for HCC may provide more survival benefits for patients. With increasing evidence of the safety and efficacy of immune therapies, such as anti-PD-1/PD-L1 and anti-CTLA-4 antibodies, in the treatment of various malignant tumours, research on the combination of immunotherapy with surgery, radiotherapy, chemotherapy, targeted therapy, and other treatment modalities in HCC is ongoing. To date, the US FDA has approved the anti-PD-1 antibody pembrolizumab as monotherapy, as well as the combination of the anti-PD-1 antibody nivolumab and the anti-CTLA-4 antibody ipilimumab for the treatment of advanced HCC patients who have previously received sorafenib (as a second-line or later-line immunotherapy) [102, 103]. Moreover, in 2020, the FDA approved the combination of the anti-PD-L1 antibody atezolizumab and the VEGF antagonist bevacizumab for the treatment of unresectable or metastatic HCC patients who have not received prior systemic therapy. Compared with sorafenib, a conventional drug for treating HCC, the combination of atezolizumab and bevacizumab significantly improved patient OS and progression-free survival (PFS) [104]. Although immunotherapy has been successfully applied as a first-line treatment for HCC, the response rate and clinical benefits of treatment are still limited due to various mechanisms of intrinsic or acquired resistance. Therefore, the combination of immunotherapy with other therapies urgently needs further research in the treatment of HCC patients [105]. Previous studies have shown that anti-CTLA-4 therapy reduces the levels of Treg cells, and anti-PD-L1 antibody reverses T-cell exhaustion, thus increasing the ratio of CD8+ T cells to Treg cells [94]. Radiotherapy amplifies the diversity of the TCR repertoire of T cells within tumours and upregulates various immune checkpoints on tumour cells. This upregulation can be overcome by immune checkpoint inhibitors; thus, the combination of radiotherapy and immunotherapy holds promise for achieving better treatment outcomes in malignant tumours [106]. Based on such promising observations, an increasing number of combinations of radiotherapy with different immunotherapies have been studied in HCC, and many have entered phase III clinical trials (Fig. 3).

A small retrospective study analysed five unresectable HCC patients who received combined treatment with SBRT and anti-PD-1 therapy. During a median follow-up period of 14.9 months, no patients experienced tumour progression, and both local control and overall survival rates were 100% at one year, demonstrating the potential value of combined treatment [107]. The immune checkpoint T-cell immunoglobulin and mucin-domain containing molecule 3 (TIM3) was upregulated in tumour-infiltrating CD8+ and CD4+ T cells [108]. The researchers compared the effects of anti-TIM3 alone or in combination with radiation therapy in a mouse HCC model, and the results showed that the combination therapy significantly slowed tumour progression and prolonged median survival compared to monotherapy. The enhanced antitumour effect of combination therapy was associated with increased tumour cell apoptosis, decreased proliferation, and reactivation of CD8+ T cells [109]. A phase I trial investigated the combination of the CTLA-4 inhibitor ipilimumab with stereotactic ablative radiotherapy (SABR) in liver cancer patients, either concurrently or sequentially with radiotherapy. The results showed significant T-cell activation in the liver after combination treatment, characterized by an increased proportion of T cells expressing ICOS, GITR, and 4-1BB [110]. A phase I study evaluated the safety of combining pembrolizumab with multisite SBRT in patients with metastatic solid tumours, and the overall objective response rate was 13.2%, with a median overall survival of 9.6 months and a median progression-free survival of 3.1 months. This suggests that pembrolizumab given after multi-site SBRT is well-tolerated and has an acceptable toxicity profile in patients with metastatic solid tumours [111].

Previous studies have found that radiotherapy induces p53-independent transcriptional upregulation of VEGF in the serum of HCC patients, increasing VEGF secretion in a dose-, time-, and cell type-dependent manner, promoting intrahepatic and extrahepatic tumour progression outside the radiotherapy area, and offsetting the benefits of radiotherapy on overall survival [112]. Dual blockade of angiogenesis and immune checkpoints has been used in the first-line treatment of HCC, so the combination of this approach with radiotherapy has the potential to provide more clinical benefits for patients [113]. On the one hand, radiotherapy can reprogram the immunologically poor "cold" TME into a more immunologically active "hot" TME [114]. On the other hand, antiangiogenic agents can promote the transport of immune effector cells to the tumour site and partially limit hypoxia through vascular normalization, driving DC maturation, reducing MDSC and Treg levels, and transiently increasing perfusion, thereby increasing the radiosensitivity of tumour cells and improving the efficiency of radiotherapy [115]. Studies have shown that sorafenib can selectively inhibit radiation-induced upregulation of VEGFR-2, induce DNA damage, and decrease DNA repair capacity, thereby enhancing the radiosensitivity of HCC [116]. Immune checkpoint inhibitors can also enhance the efficacy of antiangiogenic therapy by recruiting immune cell subtypes with vascular regulatory functions, providing a strong rationale for the development of this combination therapy [117].

The repair of DNA damage in tumour cells partially offset the therapeutic effect of radiotherapy, and combination therapy with radiotherapy, anti-PD-L1, and DNA repair inhibitors has been studied in HCC. Researchers found that the DNA repair inhibitor AZD6738 significantly increased CD8 + T-cell infiltration and activation induced by radiotherapy, resulting in a significant improvement in the tumour immune microenvironment. The antitumour efficacy and survival rate improvement of this triple combination therapy is due to the more effective activation of the cGAS/STING signalling pathway by AZD6738, which is conducive to the synergistic effect between radiotherapy and anti-PD-L1, and this triple combination therapy can generate stronger immune memory and persistent antitumour immunity, thereby preventing tumour recurrence [118]. In addition to immune checkpoints, research has also explored the use of tumour vaccines in HCC patients. A phase I study of cell-based immunotherapy using personalized peptide vaccine (PPV-DC-CTL) combined with radiotherapy for unresectable advanced HCC patients showed a remission rate of 33% and a disease control rate of 66% after radiotherapy and one to three cycles of vaccine treatment. Most patients did not experience significant haematological side effects, and no patients had liver or kidney side effects, indicating that this combination treatment regimen can provide a new, well-tolerated, safe, and effective treatment strategy for advanced HCC patients [119]. Previous studies have demonstrated that EGFR activation subsequently increases cellular radiation resistance by promoting DNA damage repair [49]. Cetuximab is an agent targeting EGFR and is currently approved for use in head and neck squamous cell carcinoma (HNSCC) and metastatic colorectal cancer; its combination with radiotherapy continues to be studied [120, 121]. A phase III trial demonstrated that radiotherapy combined with cetuximab improved overall survival in patients with locoregionally advanced squamous cell carcinoma of the head and neck (LASCCHN) at 5 years compared to that with radiotherapy alone [122]. This combination may also play an essential role in remodelling the TME. It has been shown that the combination of radiotherapy and cetuximab can increase the infiltration of NK cells and CD8 + T cells in the TME and that the combination can activate the innate antitumour immune response, which improves the outcome of HNSCC [123]. Experiments in vivo also demonstrated the promise of cetuximab alone or in combination for the treatment of HCC [124]. Thus, cetuximab in combination with radiotherapy and immunotherapy holds promise for future breakthroughs in the treatment of HCC.

Although several clinical trials involving different types of radiotherapy and tumour stages have been conducted to investigate the efficacy of various immune therapies and radiotherapy in patients with HCC, there is still a lack of prospective clinical data on the combination of these therapies. Biomarkers that can predict patient suitability and treatment outcomes still urgently need to be identified. Studies have analysed the clinical significance of soluble PD-L1 (sPD-L1) levels in HCC patients after radiotherapy. sPD-L1 levels are significantly correlated with advanced features such as tumour stage, size, and portal vein tumour thrombosis (PVTT) and are an important adverse prognostic factor for OS. Patients with higher initial sPD-L1 levels have significantly worse overall survival, and higher sPD-L1 levels one month after radiotherapy are associated with early lung metastasis. It is evident that the combination of radiotherapy and anti-PD-L1 therapy could be a promising treatment strategy for HCC, and sPD-L1 levels may serve as a potential biomarker to predict treatment efficacy [125]. In addition, researchers have developed a model to predict the response of immune checkpoint inhibitors (ICIs) combined with radiotherapy and applied this model to study combined treatment regimens for HCC patients, based on ongoing clinical trials of radioimmunotherapy. The results showed that the constructed model successfully predicted the tumour growth patterns observed in early clinical trials of monotherapy with durvalumab (anti-PD-L1 antibody) in HCC patients. Adding radiotherapy to unirradiated tumour sites on the basis of monotherapy increased the clinical benefit from 33 to 71% for 90% of irradiated tumour cells. The results of this model are consistent with clinical data, indicating that machine learning prediction models have the potential to play a valuable role in evaluating the effectiveness and applicability of radioimmunotherapy [126].

## Conclusion and perspective

The management of HCC has continually improved, particularly in the development of systemic therapies. Standard local treatments are no longer sufficient to meet clinical needs, and combination with systemic therapies is the future trend. Although radiation therapy has not yet become the standard for HCC, its excellent performance in terms of local control, overall survival, and bridging therapy as an alternative to traditional therapies has been proven. Moreover, the positive impact of radiation therapy on the immune landscape cannot be ignored. The unique immune landscape of HCC makes immunotherapy a promising treatment option for patients, and the continuous innovation of radiation therapy techniques has improved their safety and efficacy in the treatment of HCC. The combination of the two strategies could further benefit patients. However, there are currently limited studies on the combination of radiation therapy and immunotherapy in HCC, and robust prospective data are particularly lacking, which still leaves significant research gaps in this field [127]. More phase III clinical trials are needed to provide more robust evidence of efficiency and safety (Table 1). Further research is needed to clarify many key factors, such as (a) the optimal treatment sequence in combination therapy; (b) the best immunotherapy drug and the best method for degerming this; (c) strategies for determining the optimal radiation therapy dose; (d) biomarkers that can predict treatment outcomes; and (e) strategies for overcoming potential drug resistance. There are many more issues that need to be addressed, and the combination of radiation therapy and immunotherapy has shown great potential due to their synergistic effects. Therefore, conducting more research to answer these questions and promote the development of radiation-immunotherapy is of great significance.

**Table 1:** Clinical trials of radiotherapy combined with immunotherapy

Clinical trial identification(study name)	Phase	Disease	Type of radiotherapy	Type of immunotherapy	Treatment design	Estimated enrollment	Primary outcome measures
NCT04913480	II	HCC	SBRT	Durvalumab (anti-PD-L1)	SBRT → durvalumab	37	Progression-free survival (PFS)
NCT05225116	I	HCC	Radiotherapy	Sintilimab (anti-PD-1)	(Sintilimab + lenvatinib) → radiotherapy	20	Safety (number of participants with adverse events)
NCT05185531	I	HCC	SBRT	Tislelizumab (anti-PD-1)	Neoadjuvant tislelizumab + SBRT	20	ORR, pCR (pathological complete response), pPR (pathological partial response), MPR (major pathologic response)
NCT04169399	II	HCC	SBRT	Toripalimab (anti-PD-1)	Toripalimab + sBRT	30	PFS
NCT04709380	III	Advanced HCC	Radiotherapy	Toripalimab (anti-PD-1)	(Radiotherapy + toripalimab) vs sorafenib	85	Time to progression (TTP)
NCT03482102	II	HCC	Radiation	Durvalumab (anti-PD-L1) and Tremelimumab (anti-CTLA-4)	Tremelimumab + durvalumab + radiation	70	Overall response rate (ORR)
NCT03316872	II	HCC	SBRT	Pembrolizumab (anti-PD-1)	Pembrolizumab + SBRT	30	Overall response rate (ORR)
NCT05530785	II	Non-resectable HCC	Radiotherapy	Sintilimab (anti-PD-1)	radiotherapy + (sintilimab and bevacizumab biosimilar)	35	Overall response rate (ORR)
NCT04104074	I	HCC	Radiotherapy	Sintilimab (anti-PD-1)	Radiotherapy + sintilimab	20	Safety (number of participants with adverse events)
NCT03857815	II	HCC	SBRT	Sintilimab (anti-PD-1)	SBRT + sintilimab	30	PFS
NCT04857684	I	HCC	SBRT	Atezolizumab (anti-PD-L1)	SBRT + atezolizumab + bevacizumab	20	Safety (number of participants with adverse events)
NCT03203304	I	HCC	SBRT	Nivolumab (anti-PD-1) and Ipilimumab (anti-CTLA-4)	SBRT + nivolumab OR SBRT + nivolumab + ipilimumab	14	Safety (number of participants with adverse events)
NCT05096715	I	Non-resectable HCC	SBRT	Atezolizumab (anti-PD-L1)	SBRT + atezolizumab + bevacizumab	20	Dose limiting toxicity rate
NCT04611165	II	Advanced HCC	EBRT (External beam radiotherapy)	Nivolumab (anti-PD-1)	Nivolumab → EBRT	50	PFS
NCT04850157	II	HCC with portal vein tumor thrombus (PVTT)	Intensity modulated radiation therapy (IMRT)	Tislelizumab (anti-PD-1)	Tislelizumab + IMRT	30	Relapse-free survival ( RFS)
NCT04167293	II	HCC	SBRT	Sintilimab (anti-PD-1)	SBRT or SBRT + sintilimab	116	PFS
NCT05010434	II	Recurrent HCC	Radiotherapy	Sintilimab (anti-PD-1)	Radiotherapy + sintilimab + bevacizumab	46	Overall response rate (ORR)
NCT05366829	II	HCC	Radiotherapy	Toripalimab (anti-PD-1)	Radiotherapy + tislelizumab	35	Safety (number of participants with adverse events)
NCT04430452	II	Advanced HCC	Hypofractionated radiotherapy (HPT)	Durvalumab (anti-PD-L1) and Tremelimumab (anti-CTLA-4)	HPT + durvalumab ± tremelimumab	30	Overall response rate (ORR)
NCT03380130	II	HCC	Selective internal radiation therapy (SIRT) using Y-90-loaded microspheres	Nivolumab (anti-PD-1)	SIRT + nivolumab	41	Incidence of adverse event
NCT05625893	II	HCC	proton beam therapy (PBT)	Atezolizumab (anti-PD-L1)	PBT → (atezolizumab + bevacizumab)	63	Incidence of adverse event, PFS
NCT04547452	II	Stage IV HCC	SBRT	Sintilimab (anti-PD-1)	SBRT + sintilimab OR sintilimab	84	PFS
NCT05377034	II	Locally Advanced HCC	SIRT	Atezolizumab (anti-PD-L1)	SIRT + atezolizumab + bevacizumab	176	Best overall response rate (BORR)
NCT05701488	I	HCC	SIRT	Durvalumab (anti-PD-L1) and Tremelimumab (anti-CTLA-4)	Durvalumab + tremelimumab vs durvalumab + tremelimumab + SIRT	20	Safety (number of participants with adverse events)
NCT02837029	I	Stage IIIA—IVB HCC	SIRT	Nivolumab (anti-PD-1)	SIRT + nivolumab	27	Maximum tolerated dose (MTD), overall response rate (ORR)
NCT04785287	I/II	Metastatic liver carcinoma	SBRT	Nivolumab (anti-PD-1) and BMS986218 (anti-CTLA-4)	SBRT + BMS986218 ± nivolumab	13	Incidence of adverse event

## Data Availability

Not applicable.

## Abbreviations

HCC: Hepatocellular carcinoma
PD-1: Programmed cell death protein 1
PD-L1: Programmed cell death protein 1 ligand 1
CTLA-4: Cytotoxic T-lymphocyte-associated protein 4
VEGF: Vascular endothelial growth factor
SBRT: Stereotactic body radiotherapy
KCs: Kupffer cells
CCA: Cholangiocarcinoma
HBV: Hepatitis B virus
HCV: Hepatitis C virus
TACE: Transarterial chemoembolization
RFA: Radiofrequency ablation
OS: Overall survival
HPT: High-dose hypofractionated proton beam therapy
CHK1: Cell cycle checkpoint kinase 1
HIF-1α: Hypoxia inducible factor 1α
mTORC: Mammalian target of rapamycin complex
SREBP: Sterol regulatory element-binding protein
TAMs: Tumour-associated macrophages
TME: Tumour microenvironment
MDSCs: Myeloid-derived suppressor cells
Tregs: Regulatory T cells
TNF-α: Tumour necrosis factor-α
IL-1β: Interleukin-1β
CCL-2: Chemokine (C–C motif) ligand 2
CXCL10: Chemokine (C–X–C motif) ligand 10
FoxP3: Transcriptional factor P3
NKs: Natural killer cells
MHC-I: Major histocompatibility class I
DCs: Dendritic cells
TCR: T cell receptor
ICOS: Inducible co-stimulator
GITR: Glucocorticoid-induced TNF receptor (TNFR)-related protein
DDR: DNA damage repair
DSB: DNA double-strand breaks
cGAS/STING: Cyclic GMP-AMP/stimulator of interferon genes
NF-κB: Nuclear factor-κB
HR: Homologous recombination
NHEJ: Non-homologous end joining
METTL1: Methyltransferase-like 1
WDR4: WD repeat domain 4
EGFR: Epidermal growth factor receptor
RTK: Receptor tyrosine kinases
UBE2T: Ubiquitin-conjugating enzyme E2T
DNA-PKcs: DNA-dependent protein kinase catalytic subunits
SETDB1: SET domain bifurcated 1
CMA: Chaperone-mediated autophagy
HMGB1: High-mobility group box 1
PDK1: Phosphoinositide-dependent protein kinase-1
NUPR1: Nuclear protein 1
ROS: Reactive oxygen species
